# Comparative Analysis of *Luisia* (Aeridinae, Orchidaceae) Plastomes Shed Light on Plastomes Evolution and Barcodes Investigation

**DOI:** 10.3390/genes15010020

**Published:** 2023-12-22

**Authors:** Liang Ma, Cheng-Yuan Zhou, Jin-Liao Chen, Ding-Kun Liu, Siren Lan, Zhong-Jian Liu

**Affiliations:** 1 Fujian Health College, Fuzhou 350101, China; fjmaliang@126.com; 2 Key Laboratory of National Forestry and Grassland Administration for Orchid Conservation and Utilization at Landscape Architecture and Arts, Fujian Agriculture and Forestry University, Fuzhou 350002, China; zcy810338055@126.com (C.-Y.Z.); fjchenjl@126.com (J.-L.C.); fjliudk@163.com (D.-K.L.); lkzx@fafu.edu.cn (S.L.)

**Keywords:** *Luisia*, Aeridinae, Orchidaceae, plastid genome, phylogenetic analysis

## Abstract

*Luisia*, a genus of the subtribe Aeridinae of Orchidaceae, comprises ca. 40 species. Members of *Luisia* exhibit unique morphological characteristics and represent a valuable ornamental orchid genus. However, due to the scarcity of distinct morphological characters, species identification within this genus is ambiguous and controversial. In the present study, next-generation sequencing (NGS) methods were used to assemble the plastomes of five *Luisia* species and compare them with one publicly available *Luisia* plastid genome data. The plastomes of *Luisia* possessed a quadripartite structure, with sizes ranging from 146,243 bp to 147,430 bp. The plastomes of six *Luisia* species contained a total of 120 genes, comprising 74 protein-coding genes, 38 tRNA genes and eight rRNA genes. Notably, all *ndh* genes were pseudogenized or lost. An analysis of codon usage bias showed that leucine (Leu) exhibited the highest frequency, while cysteine (Cys) exhibited the lowest frequency. A total of 57 to 64 SSRs and 42 to 49 long repeats were identified. Five regions and five coding sequences were identified for DNA barcodes, based on the nucleotide diversity (Pi) analysis. The species of *Luisia* constituted a monophyletic group and were sister to *Paraphalaenopsis* with strong support. Our study deepens the understanding of species identification, plastome evolution and the phylogenetic positions of *Luisia*.

## 1. Introduction

*Luisia* Gaudichaud (1829:426) is a genus of the subtribe Aeridinae, established by Charles Gaudichaud-Beaupré in 1829, with the type species *Luisia teretifolia* Gaudich. This genus is primarily found in tropical and subtropical Asia and the Western Pacific, comprising approximately 40 species [1,2]. *Luisia* is characterized by its erect stems, terete leaves with persistent leaf bases, short racemes, and lip often distinctly divided into hypochile and epichile [3]. Due to its unique morphological characteristics and ability to easily cross with other genera, *Luisia* is well-known in worldwide horticulture and has become a valuable ornamental orchid. According to the records of the Royal Horticultural Society (http://apps.rhs.org.uk/horticulturaldatabase/orchidregister/orchidregister.asp, accessed on 30 September 2023), approximately 86 artificial intergeneric hybrids have been registered. Additionally, previous studies have reported that the leaves of *Luisia* species were used as a medicinal herb, indicating the important medicinal value of this genus [4].

Taxonomically, distinguishing and identifying *Luisia* species constitutes a significant challenge, due to the scarcity of distinct morphological characters among different species, making this genus a problematic taxonomic group [5]. Morphologically, Seidenfaden indicated that floral size was considered important evidence within *Luisia* [6]. Molecular phylogenetics, however, revealed that the phylogenetic relationships were inconsistent with the taxonomy based on floral size [7]. Additionally, the intrageneric relationships of *Luisia*, based on one of few traditional DNA sequences, commonly revealed unstable topologies with weak to moderate support [8,9]. These results showed that morphological evidence and a small number of traditional molecular markers are not sufficient for the classification of *Luisia*. Therefore, the comprehension of the phylogenetically recalcitrant group remains rudimentary.

Reliable species identification is essential for biodiversity conservation, evolutionary analysis and the protection of germplasm resources [10,11]. The selection of appropriate plant DNA molecular markers has been a topic of extensive debate, given the inherently slow rate of nucleotide evolution and the potential for complex evolutionary processes [12,13]. Especially in challenging taxonomic groups where morphological identification is difficult, the development of specific molecular markers is particularly crucial. The core DNA barcodes have been extensively utilized in most taxonomic studies [14,15] but have limitations in certain taxonomic complexity lineages of Orchidaceae [8,16]. However, the phylogenetic relationships within *Luisia*, based on the core DNA barcodes, remain uncertain, due to weak statistical support and phylogenetic discordance [1,8]. To further advance the precise identification and systematic relationships of the *Luisia* species, the specific DNA barcodes of *Luisia* need to be further assessed.

The fast advancement of next-generation sequencing (NGS) technology has significantly facilitated the acquisition of complete plastomes [17,18]. Plastomes are characterized by a high conservation of protein-coding gene structure and gene content, uniparental inheritance and a moderate mutation rate [19]. The whole plastome sequences were also known as ultra-barcodes or next-generation barcoding [20,21]. Plastome data have recently played a crucial role in advancing our understanding of phylogenetic relationships within some complex lineages of Orchidaceae [10,22]. In addition, the characterization and comparative analysis of plastomes have provided new insights into molecular evolutionary patterns, including gene duplication, loss, rearrangement and transfer [23,24,25]. However, the absence of studies on *Luisia* plastomes has significantly impeded our comprehension of the plastid genome evolution of and phylogeny of this complex group.

In the present study, we assembled and annotated complete plastid genomes for five *Luisia* species and compared them with one publicly available *Luisia* plastome. We analyzed variations in plastid genome size, gene content and structure, the contraction and expansion of inverted repeats (IR). Additionally, we examined codon-usage bias, and identified sequence divergence and variant hotspot regions. Our molecular analysis revealed the phylogenetic relationships of Aeridinae based on plastome data. This study aims to shed new light onto the understanding of plastid genome characteristics, phylogeny and evolutionary pattern of *Luisia*.

## 2. Materials and Methods

### 2.1. Taxon Sampling and Sequencing

Fresh and health leaf materials were obtained from living plants and transplanted at Fujian Agriculture and Forestry University (Fujian, China) and Shanghai Chenshan Botanical Garden (Shanghai, China). Voucher specimens were deposited in Fujian Agriculture and Forestry University (FJFC). After detailed morphological comparison, Dr. Liang Ma and Prof. Zhong-Jian Liu performed plant material identification. All plant materials were identified in their flowering period. Based on the previous study [22], a total of 37 taxa from 22 genera were sampled, which included six *Luisia* species. The voucher details and GenBank accessions for the five newly sequenced *Luisia* species are listed in Supplementary Table S1.

DNA extraction from leaf materials was carried out using the Plant Mini Kit (Qiagen, Valencia, CA, USA), following the protocol of the manufacturer. DNA degradation was assessed using 1% agarose gels. Illumina libraries were constructed according to the manufacturer’s protocol and paired-end sequencing was executed on the Illumina HiSeq 4000 system (Illumina, San Diego, CA, USA). Each species obtained approximately 10 Gb of clean data.

### 2.2. Plastid Genome Assembly and Annotation

To acquire plastid-like reads, the clean data underwent filtering using the GetOrganelle pipeline (https://github.com/Kinggerm/GetOrganelle, accessed on 30 September 2023) [26] with the following settings: −R 15, −k 21, 45, 65, 85, 105, −w 85. The filtered reads were assembled with a de novo approach using SPAdes v3.12.0 [27]. The resulting filtered De Bruijn graphs were further examined using Bandage [28].

The plastomes of *Luisia* were annotated using the Plastid Genome Annotator (PGA) [29], and the published sequence of *Paraphalaenopsis denevei* (OR159903) was used as a reference. Manual checking and adjustments, including the determination of the positions of start codons and stop codons and identification of gene pseudogenization or loss, were performed by Geneious R11.1.5 [30]. Finally, the circular plastid genome annotation maps were drawn by OGDRAW [31]. Genome sizes, GC content and the number of genes were calculated using Geneious R11.1.5 [30].

### 2.3. Codon Usage and Plastome Structure Analysis

A comprehensive set of 68 protein-coding genes (CDS) from each *Luisia* plastome, with the exception of the *ndh* genes, were extracted and concatenated by PhyloSuite v1.2.2 [32]. The computation of relative synonymous codon usage (RSCU) values was performed by DAMBE [33]. TBtools [34] was employed to generate the heatmap illustrating the RSCU values for each *Luisia* species.

We used Mauve [35] to align the plastomes of *Luisia* species for plastome rearrangement analysis, with *P. denevei* (OR159903) as the reference. The visualization of genes in the boundary regions was conducted using the IRscope program [36]. MISA (http://misaweb.ipk-gatersleben.de/, accessed on 30 September 2023) [37] was employed to detect simple sequence repeats (SSRs); settings were set according to the method previously described by Jiang et al. [25]. The REPuter [38] was employed to detect long repeat sequences, including forward (F), palindrome (P), reverse (R) and complement (C). The oligonucleotide repeats were required to have a minimum size of 30 bp, and the Hamming distance was set at 3. Visualization of the results was performed using the *ggplot2* R package [39].

### 2.4. Sequence Divergence, Barcoding Investigation and Phylogeny Reconstruction

The diversity of *Luisia* plastomes was analyzed using the Shuffle-LAGAN alignment program in the mVISTA tool [40], and *P. denevei* (OR159903) served as a reference. Nucleotide diversity (Pi) was estimated using DnaSP6 [41] with the default parameters.

The whole plastome sequences were aligned in MAFFT [42], and we employed TrimAL v1.4 [43] to remove erroneous columns in the alignments with the heuristic method. Phylogenetic trees were constructed using maximum likelihood (ML), maximum parsimony (MP), and Bayesian inference (BI) methods through the CIPRES Science Gateway web server [44,45,46]; these were performed as previously described in our previous study [10].

## 3. Results

### 3.1. Plastome Structure and Features

**Figure 1:**
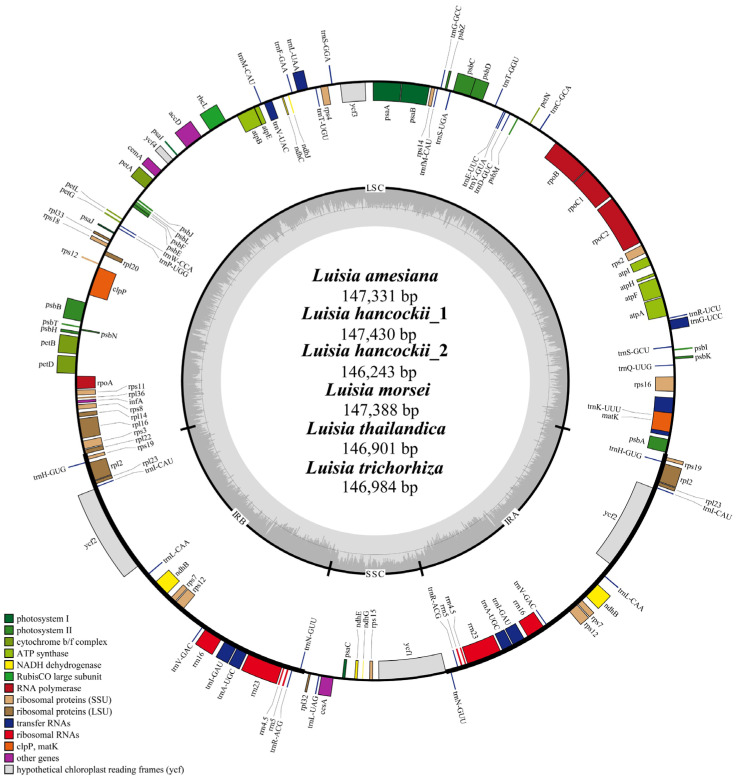
Plastid genome annotation maps of *Luisia amesiana*, *Luisia hancockii*, *Luisia hancockii* (OR030420), *Luisia morsei*, *Luisia thailandica* and *Luisia trichorhiza*. The deeper shade in the inner circle reflects the GC content. The IRA and IRB, LSC, and SSC are highlighted external to the GC content.

The sizes of plastomes in the six species of *Luisia* species varied between 146,243 bp and 147,430 bp. Each *Luisia* plastome comprised the typical quadripartite structure (Figure 1), with two invert repeat regions (IRA and IRB) (25,195–25,460 bp), a large single-copy region (LSC) (85,208–85,953 bp) and a small single-copy region (SSC) (10,195–11,412 bp). The overall GC content of all the *Luisia* plastomes was 36.6% (Table 1).

**Figure 2:**
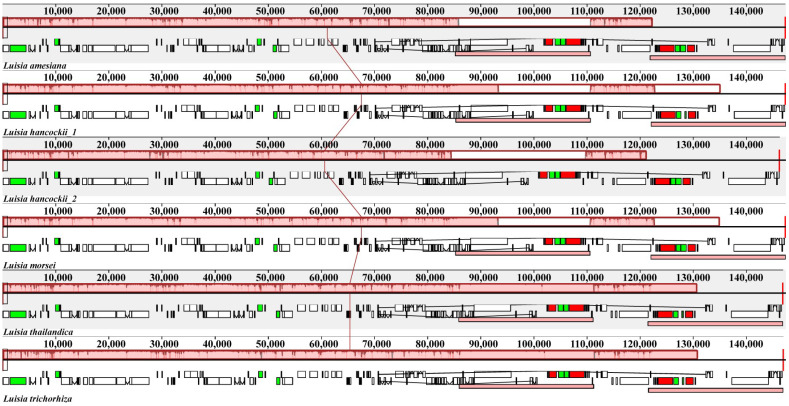
Comparative analysis of the plastomes of six species of *Luisia* using the progressive MAUVE algorithm. The locally collinear blocks are represented by blocks of the same color connected by lines. Genome regions are color-coded as CDS, tRNA, rRNA, and non-coding region.

All six *Luisia* plastomes encoded a total of 120 genes, comprising seventy-four protein-coding genes (CDS), thirty-eight transfer RNA (tRNA) genes and eight ribosomal RNA (rRNA) genes (Table 1). Among these genes, 18 genes were duplicated within the IR regions, comprising five CDSs (*rpl2*, *rpl23*, *rps7*, *rps19* and *ycf2*), eight tRNA genes (*trnA^UGC^*, *trnH^GUG^*, *trnI^CAU^*, *trnI^GAU^*, *trnL^CAA^*, *trnN^GUU^*, *trnR^ACG^* and *trnV^GAC^*), and four rRNA genes (*rrn4.5*, *rrn5*, *rrn16* and *rrn23*). All *Luisia* plastomes experienced widespread pseudogenization or the loss of *ndh* genes. The plastomes of *L. amesiana* and *L. hancockii_2* possessed six pseudogenes, *L. morsei*, *L. thailandica* and *L. trichorhiza* possessed seven pseudogenes and *L. hancockii_1* possessed nine pseudogenes. The *ndhA*, *ndhF* and *ndhH* genes were lost in six plastomes. The collinearity analysis results indicated the absence of significant rearrangements among these *Luisia* plastomes (Figure 2).

**Figure 3:**
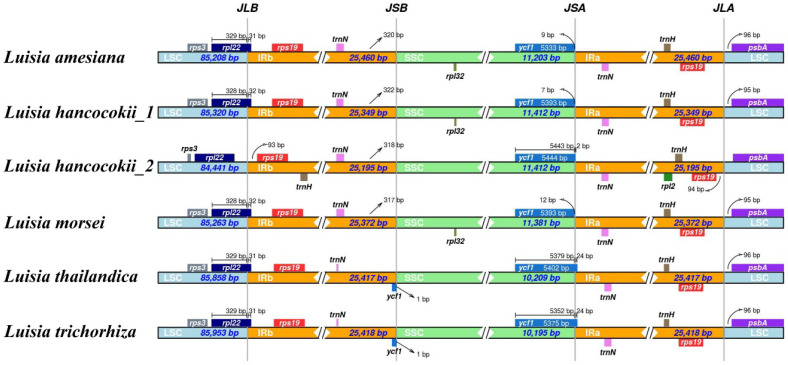
Comparative analysis of boundaries between the LSC, SSC, and IR regions among six *Luisia* plastomes.

A comprehensive comparison of the genes on the IR region boundaries was generated by comparing the six *Luisia* plastomes (Figure 3). The junctions between the IRs and the SC regions showed a high degree of conservation. At the JLB junction, the positioning of the *rpl22* gene of *L. hancockii_2* was entirely located within LSC, while in the other five species, the *rpl22* gene was spanned from LSC to IRb, covering a range of 31 to 32 bp. For the IRb/SSC (JSB) region, the boundary was situated to the right of the *trnN^GUU^* gene, with distances ranging from 317 bp to 351 bp. Additionally, the *ycf1* genes of *L. thailandica* and *L. trichorhiza* were entirely located within IRb. For the SSC/IRa (JSA) region, the *ycf1* genes of *L. hancockii_2*, *L. thailandica* and *L. trichorhiza* spanned the SSC/IRa boundary, with a range of 2 bp to 24 bp. Across all *Luisia* species, the *trnH^GUG^* and *psbA* genes were located adjacent to the junction JLA.

### 3.2. Codon Usage Analyses

**Figure 4:**
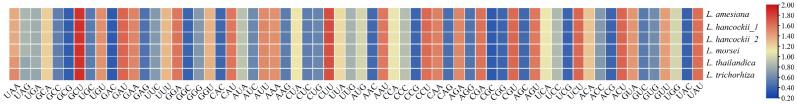
RSCU values of the codons in the six *Luisia* plastomes.

The analysis of codon usage frequency in *Luisia* plastomes utilized the concatenated matrices of 68 CDSs, excluding the *ndh* genes due to loss and pseudogenization. These CDSs were encoded by a varying range of codons, from 19,305 (*L. trichorhiza*) to 19,383 (*L. morsei*) (Supplementary Table S4). The analysis of codon usage patterns revealed a notably conserved codon usage bias (CUB) across the plastomes of the six *Luisia* species (Figure 4, Supplementary Table S4). Leu had the highest frequency among the amino acids, while Cys had the lowest frequency (Supplementary Table S4). Analysis of the relative synonymous codon usage (RSCU) showed that GCU had the highest value, averaging 1.872, while CGC exhibited the lowest average value (0.347). The highest RSCU values within the three termination codons (UAA, UAG, UGA) were observed for UAA, with a range from 1.368 to 1.500.

### 3.3. Analysis of Sequence Repeats

**Figure 5:**
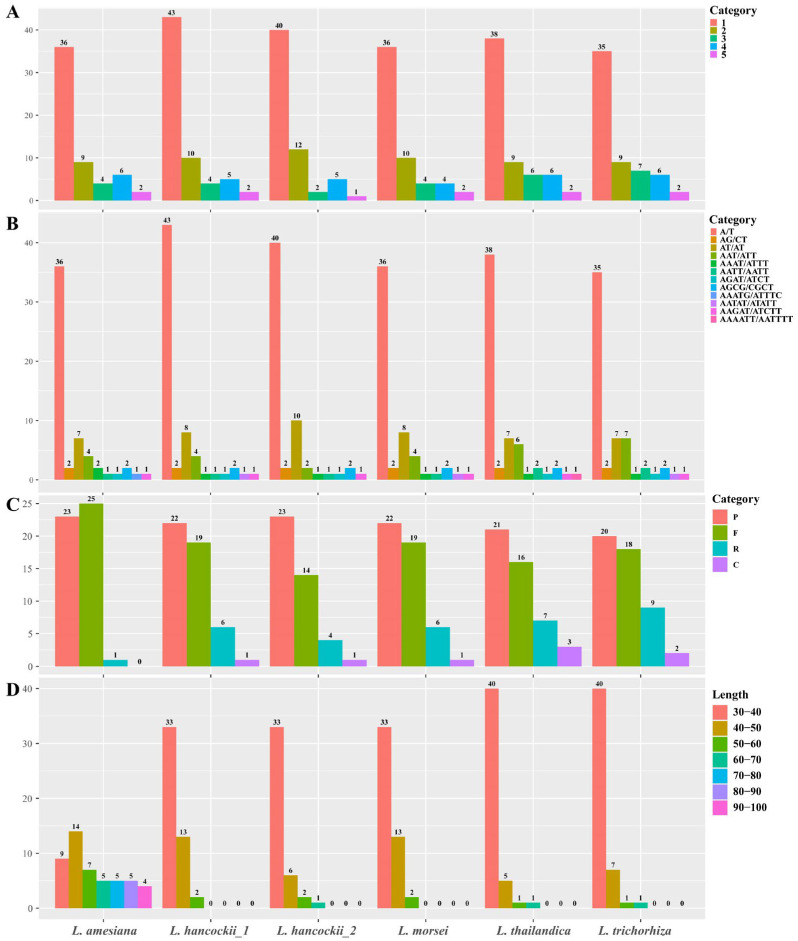
Overview of sequence repeats across the *Luisia* plastomes. (**A**) Quantity and types of SSRs; (**B**) occurrence frequency of categorized repeat types; (**C**) diversity in both abundance and type of repeats; (**D**) number of long repeats sequences categorized by length.

Different types of SSR and long repeats were examined to clarify intragenus variations. Firstly, six types of SSR were examined in *Luisia* plastomes, revealing a total of 57 SSRs in *L. amesiana* to 64 SSRs in *L. hancockii_1* (Figure 5, Supplementary Table S2). Mononucleotide repeats were the most frequent type [35,36,37,38,39,40,41,42,43], followed by dinucleotide repeats [9,10,11,12]. Hexanucleotide repeats were not detected in all *Luisia* plastomes. Additionally, it was observed that only A/T repeats [36,37,38,39,40,41,42,43] were detected in mononucleotide repeats.

All four types of long repeats were also identified in *Luisia* plastomes, with a total of 42 (*L. hancockii_2*) to 49 (*L. amesiana* and *L. trichorhiza*) long repeats detected (Figure 5, Supplementary Table S3). All four types of long repeats were present in species other than *L. amesiana*, which possessed three types (palindrome, forward and reverse). The majority of repeat sequences fell within the 30–40 bp range, with the exception of *L. amesiana*, which possessed a total of 40 repeat sequences longer than 40.

### 3.4. Plastome Sequence Divergence and Barcoding Investigation

**Figure 6:**
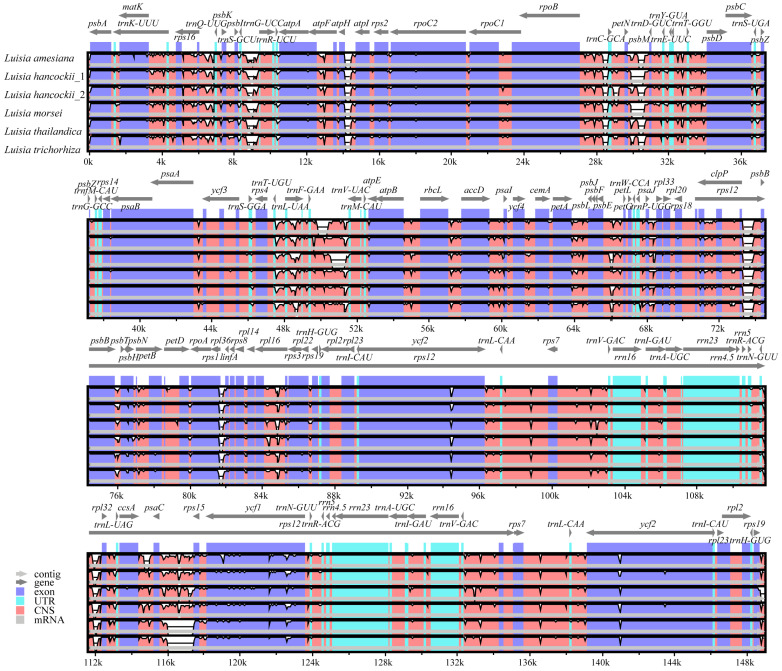
Global alignment of the six *Luisia* plastomes was conducted using mVISTA, with *P. denevei* serving as the reference.

The mVISTA platform was employed to find highly variable regions between six *Luisia* plastomes and the reference (*P. denevei*). The findings showed a notable conservation in the coding region, compared with the noncoding region (Figure 6). The most significant variation was found in the LSC regions of plastomes in *Luisia* and *P. denevei*, followed by the LSC region and IR regions (Figure 6). These findings suggest the existence of multiple intergenic or intragenic regions well-suited for DNA barcodes, enabling the effective discrimination of different *Luisia* species.

**Figure 7:**
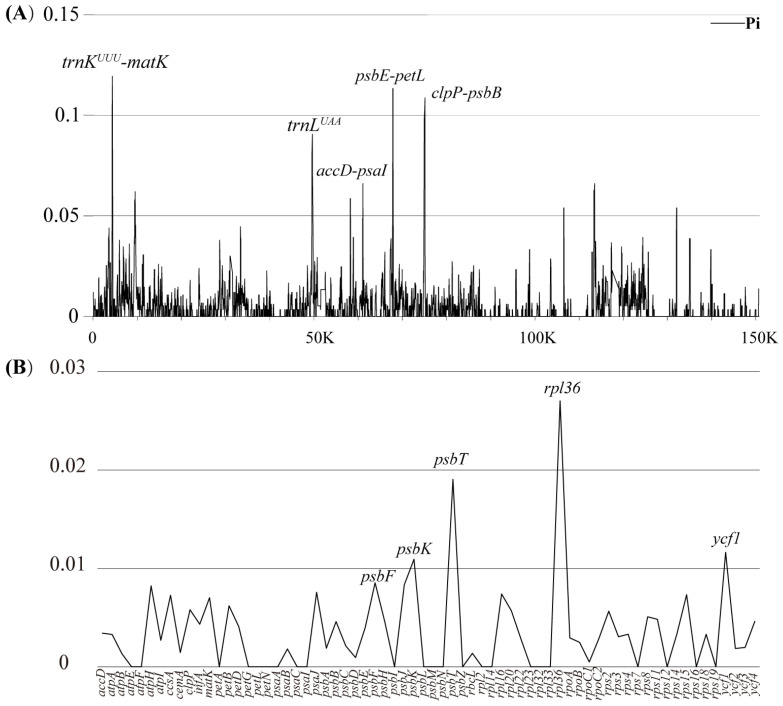
Nucleotide variability values (Pi) of six *Luisia* plastomes. (**A**) Pi values for the whole plastome with annotations indicating five mutation hotspot regions. (**B**) Pi values for 68 CDSs. The window size was configured at 100 bp, and the sliding windows size was set to 25 bp.

In order to further explore the DNA barcodes for *Luisia* plastomes, we employed DnaSP6 to calculate the Pi values based on the matrices of six *Luisia* plastomes. The findings revealed the substantial divergence of two SC regions and a conservative of IR regions (Figure 7, Supplementary Table S5). Based on the Pi values ranking, five regions, including *trnK^UUU^*-*matK*, *psbE*-*petL*, *clpP*-*psbB*, *trnL^UAA^* and *accD*-*psaI*, were identified for candidate barcodes (Figure 7A). In addition, the results highlighted five coding sequences (*rpl36*, *psbT*, *ycf1*, *psbK* and *psbF*) with high nucleotide diversity, making them suitable for phylogenetic analysis (Figure 7B).

### 3.5. Phylogenetic Analysis

**Figure 8:**
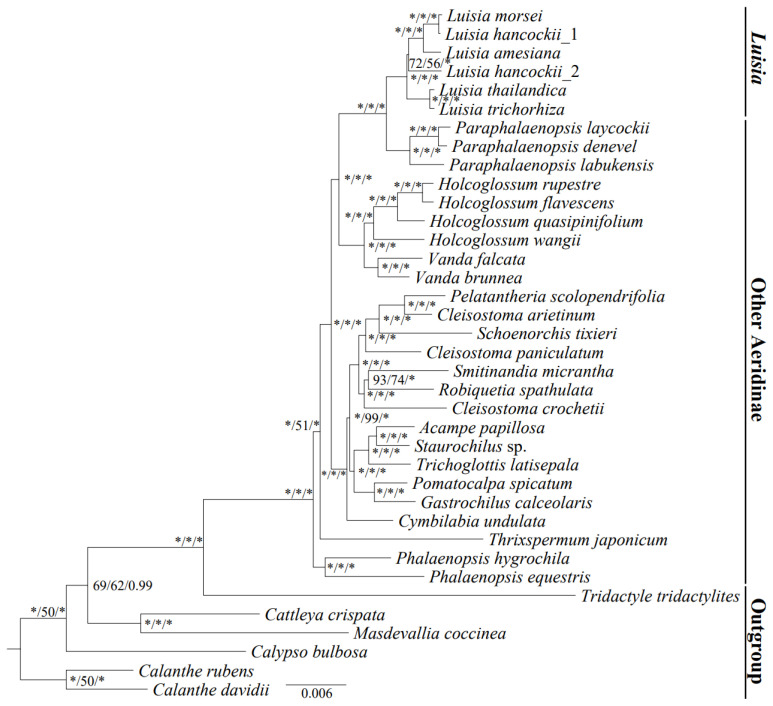
Phylogenetic tree constructed by ML analysis using the whole plastome dataset. Bootstrap percentages and Bayesian posterior probabilities (BP_ML_, BP_MP_, PP) are indicated near the nodes. * denotes nodes with a 100 bootstrap percentage or 1.00 posterior probability. The scale bar shows the number of substitutions per sites.

Phylogenetic trees were generated using three methods based on complete plastid genome and 68 CDSs, resulting in a similar topology and strong support (Figure 8, Supplementary Figure S1). The whole plastome matrix consisted of 25,852 variable sites (VS) and 11,774 parsimony informative sites (PIS). The CDS concatenated matrix consisted of 7039 VS and 3164 PIS. The phylogenetic trees showed that the species of *Luisia* formed a well-supported monophyletic group (BS = 100, PP = 1.00) and was sister to *Paraphalaenopsis*, with strong support (BS = 100, PP = 1.00). The intrageneric relationships within *Luisia* revealed that this genus could be divided into two diverging lineages with strong support (BS = 100, PP = 1.00). The taxa *L. morsei*, together with *L. hancockii_1* and *L. amesiana*, formed the first lineage, while the other species clustered into the second lineage. The analyses collectively support that *L. hancockii_1* is sister to *L. morsei*, rather than the previously published plastome of *L. hancockii_2*.

To further explore specific DNA barcodes for *Luisia*, we employed both the concatenation matrix of five informative regions and the concatenation matrix of five protein-coding sequences to reconstruct the phylogenetic relationships (Supplementary Figure S2). The phylogenetic tree inferred by informative regions showed high support values but exhibited different relationships of *Luisia hancockii_2*. The phylogenetic tree inferred by five protein-coding sequences presents the same topology, compared with the phylogenetic trees inferred by complete plastomes and 68 protein-coding genes, with strong support (BS_ML_ > 84).

## 4. Discussion

### 4.1. The Plastome Characteristics and Structural Evolution

A total of five *Luisia* plastomes were newly reported and compared with the previously reported plastome of *L. hancockii_2* (OR030420). All *Luisia* plastomes possess the typical quadripartite structure of angiosperm plastomes, consisting of a pair of IRs ranging from 25,195 to 25,460 bp, separated by the LSC (85,208–85,953 bp) and SSC (10,195–11,412 bp) regions (Figure 1). The plastome size (ranging from 146,243 to 147,430 bp) and GC content (36.6%) were similar to those observed in other Orchidaceae lineages [47,48]. Additionally, there were no rearrangements among *Luisia* plastomes detected by the collinearity analysis (Figure 2). These findings suggest a high degree of conservation among the six *Luisia* plastomes, concerning genome size, GC content, and gene order.

Our genome annotation results showed that *Luisia* plastomes encoded 120 genes, comprising 74 CDSs, 38 tRNA genes, and 8 rRNA genes, with the pseudogenization or loss of all *ndh* genes (Figure 1, Table 1), similar to other lineages of the subtribe Aeridinae [49,50]. The occurrence of pseudogenization and loss of *ndh* genes were commonly observed in some orchid groups [51,52], especially in some epiphytic lineages [10,23,53,54]. Previous studies have suggested a potential association between the pseudogenization and loss of *ndh* genes and epiphytic habitats [55]. In this study, members of *Luisia* are typically epiphytic or lithophytic [7], supporting an association between the epiphytic lifestyle of Orchidaceae and the pseudogenization and loss of *ndh* genes.

The variations in plastome length and gene content are significantly influenced by the expansion or contraction of the inverted repeat (IR) regions [56]. In this study, a slight difference in the gene arrangement of the IR/SC boundary was observed (Figure 3). For the LSC/IRb (JLB) region, the *rpl22* gene of *L. hancockii_1* was entirely located within LSC. For the SSC/IRa (JSA) region, the *ycf1* genes of *L. hancockii_2*, *L. thailandica* and *L. trichorhiza* spanned the SSC/IRa boundary. Therefore, IR/SC boundary shift might contribute to variations of plastome length in *Luisia*.

Codon usage bias plays a crucial role in shaping the evolutionary trajectories of plastid genomes, influencing the expression of gene functions. RSCU values provide valuable insights for exploring the evolutionary patterns of species [57]. Previous studies on codon usage bias in Orchidaceae revealed that species with close relationships exhibited similar codon usage bias [25,58]. Our results indicated that codon usage bias was highly conserved among *Luisia* plastomes (Figure 4, Supplementary Table S4). In the present study, Leu displayed the highest frequency, while Cys had the lowest frequency. This trend is aligning with observations in previous investigations into codon preference of other orchid lineages [25,58].

### 4.2. Phylogenetic Analysis

The low morphological variation among *Luisia* species makes this genus one of the most taxonomically complex groups within Aeridinae [6]. Based on a few traditional molecular markers, previous phylogenetic analyses showed inconsistencies in the intergeneric phylogenetic position of *Luisia*. Kocyan et al. [9] indicated that *Luisia* had a sister-group relationship with *Holcoglossum*, while Zou et al. [8] supported the theory that *Luisia* was sister to *Paraphalaenopsis* with strong support. Additionally, the intrageneric relationships within *Luisia* remained ambiguous and controversial, due to unstable topologies with weak support [1,8,9]. Here, we reconstructed the phylogenetic relationships of *Luisia* based on plastome sequences, with the aim of recovering robust phylogenetic relationships with *Luisia*. Our results showed that species of *Luisia* formed a monophyletic group and were sister to *Paraphalaenopsis* with strong support in all phylogenetic trees (BS = 100, PP = 1.00) (Figure 8, Supplementary Figure S1), consistent with the results of Zou et al. [8]. In terms of the intrageneric relationships, six *Luisia* species could be divided into two diverging lineages with strong support (BS = 100, PP = 1.00). This result indicated that the plastome sequences were ideal molecular markers for resolving the relationships of *Luisia*. Interestingly, we observed phylogenetic position incongruence between the previously published plastomes of *L. hancocki_2* (OR030420) and *L. hancocki_1*. This result reflected a potential sample misidentification of *Luisia* species by previous research, indicating the challenges in identifying *Luisia* species. Overall, our DNA molecular systematic study provides a valuable framework for understanding the systematic evolution of *Luisia.*

### 4.3. Barcoding Investigation

In the field of taxonomy, morphological characteristics are undoubtedly crucial. However, the morphological differences among species in some taxonomically complex groups are difficult to thoroughly distinguish. Therefore, the development of DNA barcoding for the identification of these species becomes particularly important. DNA barcoding has been widely used in identifying complex species [59,60] and detecting misidentifications [61]. This comparative plastomic method has been used with some lineages of Orchidaceae [23,25,49]. To investigate the specific DNA barcodes in *Luisia*, we conducted nucleotide diversity analyses of the entire plastid genome and 68 protein-coding genes. A total of five hotspots regions (*trnK^UUU^*-*matK*, *psbE*-*petL*, *clpP*-*psbB*, *trnL^UAA^* and *accD*-*psaI*) and five protein-coding genes (*rpl36*, *psbT*, *ycf1*, *psbK* and *psbF*) were selected for candidate barcodes, respectively. Based on informative regions and coding sequences, we reconstructed the phylogenetic relationships of *Luisia*, which both showed high support values but resulted in different phylogenetic positions for *Luisia hancockii_2* (Supplementary Figure S2). When facing challenges in the morphological identification of *Luisia* species, these hotspot molecular markers could provide rapid and accurate molecular identification. The DNA barcodes explored in this study, based on plastome data, provide an insightful window into the investigation of *Luisia* species identification.

In addition, previous studies indicated that SSRs and long repeats are valuable for species identification and the development of molecular markers [62,63]. Our results showed that most of the long repeat sequences in *Luisia* plastomes fell within the range of 30 to 40 bp, which is consistent with previous studies in Orchidaceae [23,24,25]. However, we observed that the plastome of *L. amesiana* possessed a total of 40 repeat sequences longer than 40, indicating that these sequences could potentially serve as specific DNA barcodes for this species. Our findings significantly contribute to advancing the development of precise DNA barcodes designed specifically for the *Luisia*.

## 5. Conclusions

A comparative examination of six complete plastid genomes of *Luisia*, five of which were newly sequenced in this investigation, has provided insights into their structural organization and sequence evolution. The genomic characteristics, features, gene content and gene order of *Luisia* plastomes exhibit a high level of conservation. Loss or pseudogenization was observed in all *ndh* genes. Based on plastome data, phylogenetic analysis was performed to identify the intrageneric and intergeneric relationships of *Luisia* and found that the plastome sequences were powerful tools for unraveling the relationships of *Luisia*. Several highly variable regions and genes were identified. These highly variable loci were further used to reconstruct the phylogenetic relationships of *Luisia*, revealing stable topologies with strong support. Therefore, our studies advance our understanding of the characteristics and evolutionary patterns of *Luisia* plastomes, shedding light on DNA barcoding investigation and molecular identification for *Luisia* species conservation.

## Figures and Tables

**Table 1:** Characteristics of the six complete plastomes of *Luisia*.

Scientific Name	Size (bp)	GC (%)	LSC (%)	IR (%)	SSC (%)	Total Number of Genes	Protein-Encoding Gene	tRNA	rRNA	Number of *ndh* Fragments
*L. amesiana*	147,331	36.6	85,208 (57.83)	25,460 (17.28)	11,203 (7.60)	120	74	38	8	6
*L. hancockii_1*	147,430	36.6	85,321 (57.87)	25,348 (17.19)	11,412 (7.74)	120	74	38	8	9
*L. hancockii_2*	146,243	36.6	84,438 (57.74)	25,195 (17.23)	11,412 (7.80)	120	74	38	8	6
*L. morsei*	147,388	36.6	85,263 (57.85)	25,372 (17.21)	11,381 (7.72)	120	74	38	8	7
*L. thailandica*	146,901	36.6	85,858 (58.45)	25,417 (17.30)	10,209 (6.95)	120	74	38	8	7
*L. trichorhiza*	146,984	36.6	85,953 (58.48)	25,418 (17.29)	10,195 (6.94)	120	74	38	8	7

## Data Availability

The plastome sequences are deposited in GenBank at the NCBI repository, accession numbers OR948794–OR948798.

## Supplementary Materials

The following supporting information can be downloaded at: https://www.mdpi.com/article/10.3390/genes15010020/s1, Supplementary Figure S1: Phylogenetic tree obtained by maximum-likelihood analysis based on 68 protein coding-genes. The numbers near the nodes are bootstrap percentages and Bayesian posterior probabilities (BP_ML_, BP_MP_, PP). A dash (-) indicates that a node is inconsistent between the topology of the MP/ML trees and the Bayesian tree; *node is 100 bootstrap percentage or 1.00 posterior probability; Supplementary Figure S2: Phylogenetic tree obtained by maximum-likelihood analysis based on informative regions (A) and coding sequences (B); Supplementary Table S1: Source and voucher information for this study. Voucher specimens were deposited in the herbariums of Forestry College of Fujian Agriculture and Forestry University (FJFC) and National Center for Biotechnology Information (NCBI); Supplementary Table S2: The details information of simple sequence repeats (SSRs); Supplementary Table S3: The details information of long repeats; Supplementary Table S4: The details information of relative synonymous codon usage (RSCU); Supplementary Table S5: The nucleotide diversity of plastome and 68 protein coding genes in *Luisia*.
