# Supplementary material for: Comparative Analysis of Luisia (Aeridinae, Orchidaceae) Plastomes Shed Light on Plastomes Evolution and Barcodes Investigation
Source: Genes (Basel). 2023 Dec 22;15(1):20. doi: 10.3390/genes15010020 (PMC10815154; doi:10.3390/genes15010020)
Supplement: Supplementary file 1 [file genes-15-00020-s001.zip › Supplementary Figure.pdf]

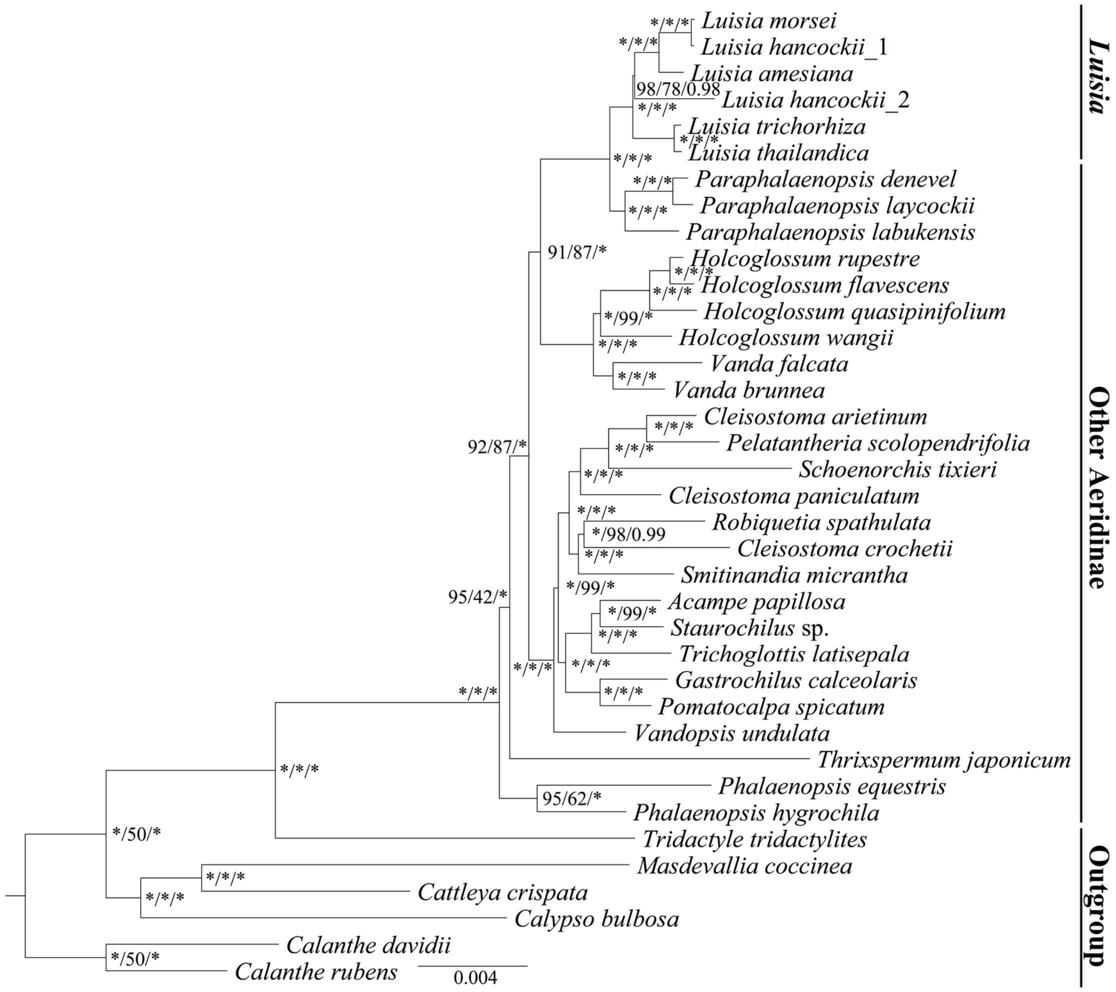

Supplementary Figure S1. Phylogenetic tree obtained by maximum-likelihood analysis based on 68 protein coding-genes. The numbers near the nodes are bootstrap percentages and Bayesian posterior probabilities (BP_{ML}, BP_{MP}, PP). A dash (-) indicates that a node is inconsistent between the topology of the MP/ML trees and the Bayesian tree; *node is 100 bootstrap percentage or 1.00 posterior probability.

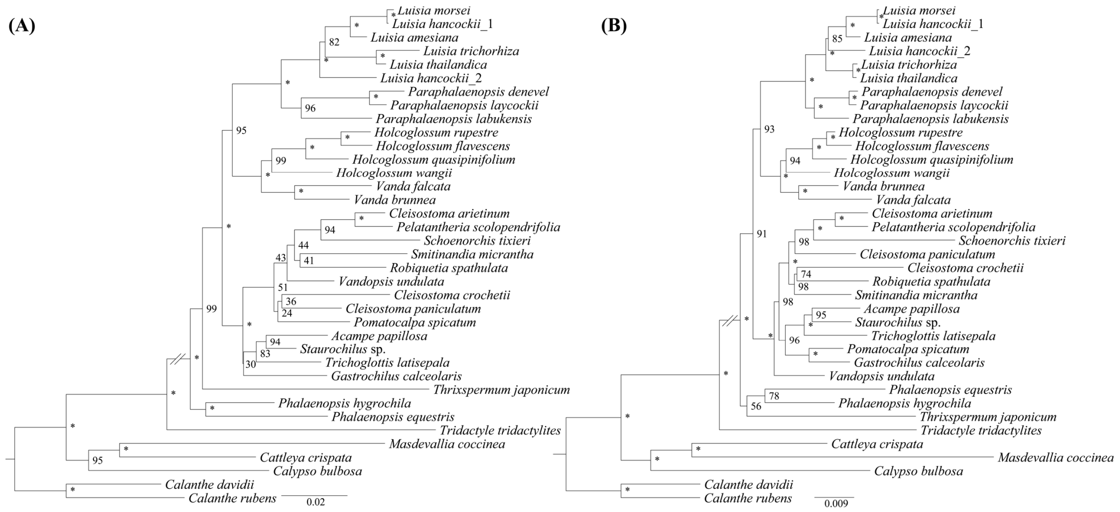

Supplementary Figure S2. Phylogenetic tree obtained by maximum-likelihood analysis based on informative regions (A) and coding sequences (B).

Supplementary Table S1. Source and voucher information for this study. Voucher specimens were deposited in the herbariums of Forestry College of Fujian Agriculture and Forestry University (FJFC) and National Center for Biotechnology Information (NCBI).

Supplementary Table S2. The details information of simple sequence repeats (SSRs).

Supplementary Table S3. The details information of long repeats.

Supplementary Table S4. The details information of relative synonymous codon usage (RSCU).

Supplementary Table S5. The nucleotide diversity of plastome and 68 protein coding genes in *Luisia*.
